# RPS23RG1 reduces Aβ oligomer-induced synaptic and cognitive deficits

**DOI:** 10.1038/srep18668

**Published:** 2016-01-06

**Authors:** Li Yan, Yaomin Chen, Wubo Li, Xiumei Huang, Hedieh Badie, Fan Jian, Timothy Huang, Yingjun Zhao, Stanley N. Cohen, Limin Li, Yun-wu Zhang, Huanmin Luo, Shichun Tu, Huaxi Xu

**Affiliations:** 1 Department of Pharmacology, School of Medicine, Jinan University, Guangzhou 510632, China; 2 Neuroscience and Aging Research Center, Sanford-Burnham-Prebys Medical Discovery Institute, La Jolla, CA 92037, USA; 3 Functional Genetics, Inc., Gaithersburg, MD, USA; 4 Department of Genetics, Stanford University School of Medicine, Stanford, CA 94305, USA; 5 Fujian Provincial Key Laboratory of Neurodegenerative Disease and Aging Research, Institute of Neuroscience, College of Medicine, Xiamen University, Xiamen 361102, China

## Abstract

Alzheimer’s disease (AD) is the most common form of dementia in the elderly. It is generally believed that β-amyloidogenesis, tau-hyperphosphorylation, and synaptic loss underlie cognitive decline in AD. *Rps23rg1*, a functional retroposed mouse gene, has been shown to reduce Alzheimer’s β-amyloid (Aβ) production and tau phosphorylation. In this study, we have identified its human homolog, and demonstrated that RPS23RG1 regulates synaptic plasticity, thus counteracting Aβ oligomer (oAβ)-induced cognitive deficits in mice. The level of *RPS23RG1* mRNA is significantly lower in the brains of AD compared to non-AD patients, suggesting its potential role in the pathogenesis of the disease. Similar to its mouse counterpart, human RPS23RG1 interacts with adenylate cyclase, activating PKA/CREB, and inhibiting GSK-3. Furthermore, we show that human RPS23RG1 promotes synaptic plasticity and offsets oAβ-induced synaptic loss in a PKA-dependent manner in cultured primary neurons. Overexpression of *Rps23rg1* in transgenic mice consistently prevented oAβ-induced PKA inactivation, synaptic deficits, suppression of long-term potentiation, and cognitive impairment as compared to wild type littermates. Our study demonstrates that RPS23RG1 may reduce the occurrence of key elements of AD pathology and enhance synaptic functions to counteract oAβ-induced synaptic and cognitive deficits in AD.

Alzheimer’s disease (AD) is the most common form of dementia in the elderly and is characterized pathologically by the extracellular deposition of β-amyloid (Aβ) peptides and intracellular tangles comprising phosphorylated tau proteins^1^,^2^. Soluble oligomeric Aβ species are believed to cause synaptic and neuronal loss in AD, and their expression in AD-affected regions correlates with the severity of premortem dementia more closely than the presence of insoluble Aβ plaques^3^,^4^,^5^. Aβ peptides vary in length and are derived from sequential cleavage of the β-amyloid precursor protein (APP) by β- and γ-secretases^6^,^7^. Aβ_1-42_ is the most amyloidogenic Aβ species and its oligomeric assemblies are considered most likely to induce synaptic/neuronal loss and memory decline in AD^2^,^8^.

Activation of cyclic AMP (cAMP)-dependent protein kinase A (PKA) is generally important for synaptic function and learning and memory^9^, and plays a seminal role in Aβ-associated synaptic loss and memory deficits. PKA activity has been reported to be depleted in the cortex of AD patients^10^, and is reduced in mouse brain tissues exposed to Aβ or in *APP/PS1* AD mouse brain. Restoring cAMP signaling and PKA activity can reduce AD-like phenotypes in model mice^11^,^12^. Remarkably, PKA-dependent phosphorylation of the transcription factor CREB is attenuated by Aβ application, and can be restored by pharmacological agents that upregulate cAMP/PKA signaling^11^,^12^,^13^.

The microtubule-associated protein tau has been implicated in Aβ-induced synaptic loss^14^,^15^. Application of synthetic and AD patient-derived Aβ oligomers (oAβs) can cause tau hyperphosphorylation and synaptic damage in cultured neurons, which can be inhibited by tau reduction or by blocking tau phosphorylation^16^,^17^,^18^. Tau kinases such as GSK-3α/β have also been implicated to drive AD pathogenesis, as their activities are aberrantly upregulated in AD patients and animal models^19^,^20^. PKA can phosphorylate and inactivate GSK-3α/β^21^, further suggesting that PKA may have a neuroprotective role in AD. Thus, GSK-3α/β inhibition and the resultant inhibition of tau hyperphosphorylation are important for PKA’s protective effects against synaptic and behavioral deficits in AD.

We have previously identified four mouse *Rps23rg1* (formerly *Rps23r1*) gene family members, which regulate Aβ levels and tau phosphorylation in mice^22^,^23^. No human homolog has been reported in the literature or described in public genomic databases. In the current study, we have identified the human homolog of *Rps23rg1*. We found that human and mouse genes shared about 60% homology. Notably, human *RPS23RG1* mRNA was decreased in postmortem brains of AD patients. We further demonstrated that exogenous expression of RPS23RG1 increased synapse numbers to offset oAβ-induced synaptic loss in a PKA-dependent manner in cultured neurons. Finally, transgenic overexpression of mouse Rps23rg1 *in vivo* restored PKA activity, and mitigated oAβ-induced synaptic loss, long-term potentiation (LTP) suppression, and cognitive impairment. Collectively, our results strongly suggest that upregulating RPS23RG1 and its downstream pathways may be a potential therapeutic approach for treating AD.

## Results

### Identification and characterization of the human *RPS23RG1* gene

**Figure 1:**
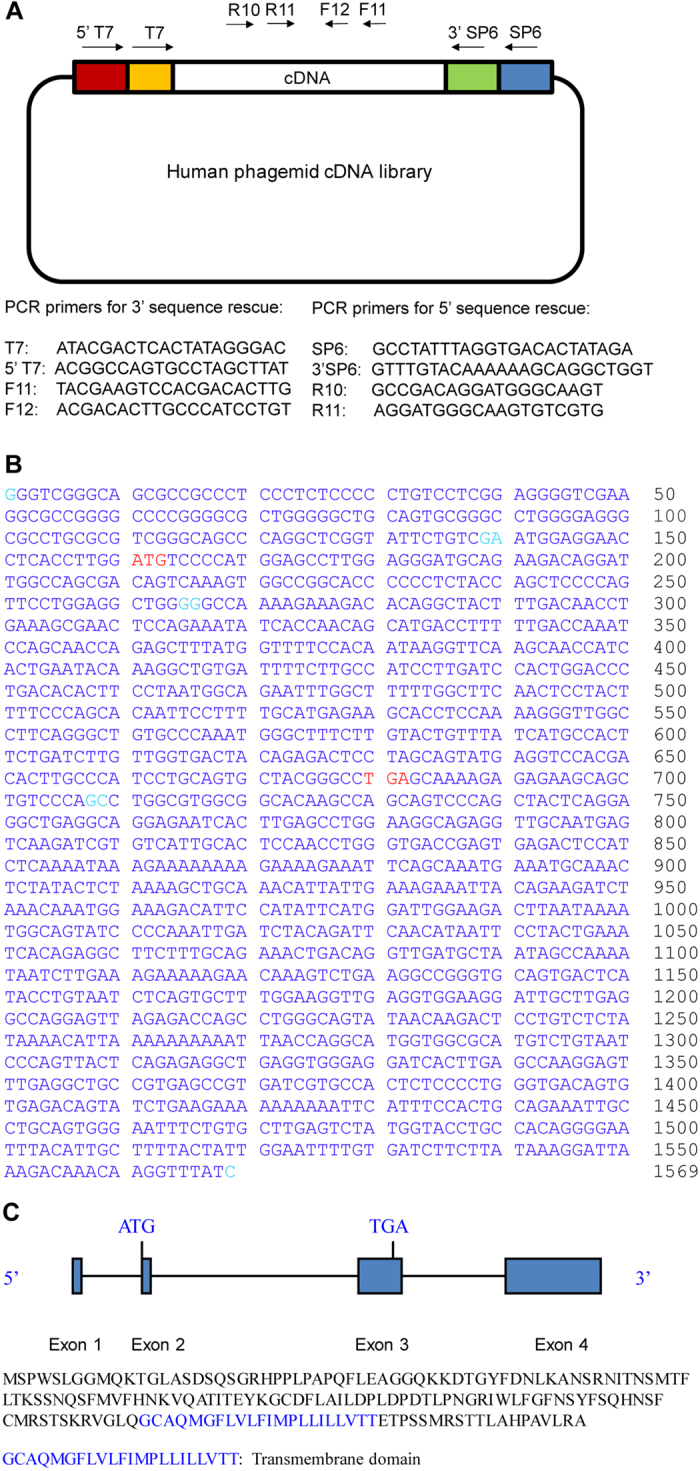
Identification and cloning of the human *RPS23RG1*gene. (**A**) Diagram showing human fetal brain cDNA library constructed in a phagemid vector. PCR primers used to amplify human *RPS23RG1* gene are depicted by black arrows. (**B**) The full-length 1569bp human *RPS23RG1* cDNA sequence was reconstituted from PCR products amplified from a human fetal brain phagemid cDNA library. The cDNA sequence was also confirmed by RT-PCR from total human fetal brain RNA. ATG start codon and TGA stop codon were shown in red. Intron and exon boundaries were shown in green. (**C**) The genomic contig of human *RPS23RG1* in chromosome 8 and the encoded protein was shown. The transmembrane domain was indicated in blue.

We searched the human genome database (http://blast.ncbi.nlm.nih.gov) for candidate human homologs of mouse *Rps23rg1*^22^,^23^, and found a region in human chromosome 8 (8q22) sharing high homology with the mouse *Rps23rg1* coding sequence. 5′ and 3′ cDNA fragments were amplified by nested PCR from a human fetal brain cDNA library constructed in a phagemid vector, using human-specific primers and primers targeting the phagemid vector sequence (Fig. 1A). A 1569-bp cDNA was isolated from the PCR products (Fig. 1B) and confirmed by RT-PCR using total RNA from human fetal brain.

Sequence analysis of the cloned cDNA suggested that the human *RPS23RG1* gene on chromosome 8 contains 4 exons spanning a 6.7 kb genomic contig. The first exon is located within a CpG island region. An open reading frame encoding a 173 amino acid protein is located within exon 2 and 3 (Fig. 1C). Human RPS23RG1 protein shares about 60% homology to its mouse homolog (Supplementary Fig. S1A). Similar to mouse Rps23rg1, the human homolog is a type 1b transmembrane protein as predicted by bioinformatic analysis (Supplementary Fig. S1B).

### Human *RPS23RG1* mRNA levels are decreased in Alzheimer’s disease

**Figure 2:**
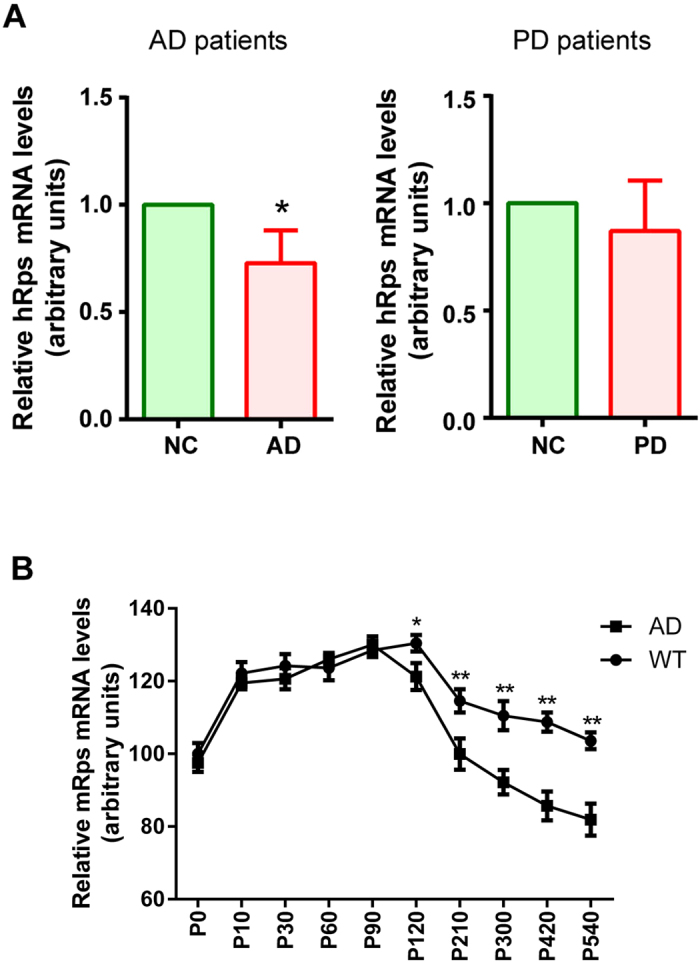
*PRS23RG1/Rps23rg1* mRNA levels are decreased in postmortem human AD patient and AD transgenic mouse brains. (**A**) Quantification of *RPS23RG1* (hRps) mRNA levels in the postmortem brains of human AD patients (left) or PD patients (right). Values were mean ± SEM (n = 8 for AD and n = 7 for PD, *p < 0.05 two-tailed Student’s *t* test). (**B**) The mRNA level of mouse *Rps23rg1* (mRps) in Tg2576 AD or WT mouse brains at different ages were quantified for comparison (n = 4 at each data point, *p < 0.05, **p < 0.01, two-tailed Student’s *t* test).

To study whether human RPS23RG1 may be involved in AD, we checked and found that *RPS23RG1* mRNA levels in frontal cortex were significantly reduced in brain samples from AD patients (Supplementary Table S1) but not from Parkinson’s disease patients (Fig. 2A), suggesting that the reduction of *RPS23RG1* expression may be specific to AD. We further measured *Rps23rg1* mRNA levels in Tg2576 mice expressing the human APP695 “Swedish” variant (KM670/671NL)^24^, and their wild type littermates. Despite similar expression at younger stages (≤P90), murine *Rps23rg1 *mRNA levels decreased from postnatal day 120 in both genotypes, but more dramatically in Tg2576 mice compared to wild type littermates (Fig. 2B).

### Human RPS23RG1 overexpression reduces Aβ levels and tau-phosphorylation by interacting with adenylate cyclase 8 and activating PKA

**Figure 3:**
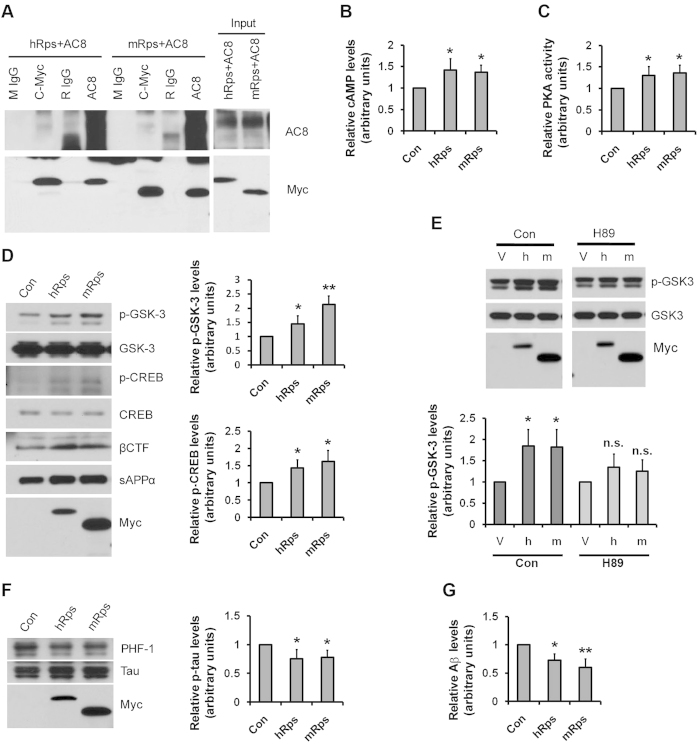
Human RPS23RG1 overexpression reduces Aβ levels and tau-phosphorylation via its interaction with adenylate cyclase 8 and consequent PKA activation. (**A**) Cells transfected with adenylate cyclase 8 (AC8) together with human RPS23RG1 (hRps) or mouse Rps23rg1 (mRps) were used for co-immunoprecipitation experiments. Cell lysates were incubated with mouse IgG (mIgG), anti-c-myc, rabbit IgG (rIgG), or anti-AC8. Immunoprecipitated proteins were subjected to western blot analysis using antibodies against AC8 or myc (for hRps or mRps, respectively). Co-IP experiments were reproduced 4 times. (**B,C**) Cells transfected with hRps, mRps, or control vector (Con) were analyzed for cAMP levels (**B**) or *in vitro* PKA activity (**C**). (**D**) Cells were transfected with hRps, mRps, or control vector (Con). Cell lysates were analyzed for phosphorylated and total GSK-3, phosphorylated and total CREB, CTF, and Rps (myc) levels. Conditioned media were analyzed using the 6E10 antibody to detect sAPPα levels. Summary graph showed relative p-GSK-3 and p-CREB levels. (**E**) Cells transfected with hRps (h), mRps (m), or control vector (V) were treated with DMSO (Con) or the PKA inhibitor H89. Cell lysates were analyzed for phosphorylated and total GSK-3 and Rps (myc) levels. Summary graph show relative p-GSK-3 levels. (**F**) Cells were transfected with hRps, mRps, or control vector (Con). Cell lysates were analyzed for phosphorylated (PHF-1 or p-tau) and total (tau) tau and Rps (myc). Summary graph showed relative p-tau levels normalized against total tau levels. (**G**) Conditioned media were also analyzed for Aβ42 secretion by ELISA as shown. All data were normalized to control values (as one arbitrary unit) and shown as mean ± SEM (n = 3, *p < 0.05, **p < 0.01, n.s.: not significant, one-way ANOVA with Dunnett’s multiple comparisons).

We have previously shown that mouse Rps23rg1 (mRps) interacts with adenylate cyclase 8^22^,^23^. To determine whether human RPS23RG1 protein (hRps) can also interact with AC8, we performed co-immunoprecipitation experiments in cultured HEK293T cells co-transfected with adenylate cyclase 8 and human RPS23RG1, mouse Rps23rg1, or control vectors. Similar to mouse Rps23rg1, human RPS23RG1 interacted with adenylate cyclase 8 (Fig. 3A). Moreover, overexpression of RPS23RG1 significantly upregulated cAMP levels (Fig. 3B) and enhanced PKA activity (Fig. 3C). Therefore, both human and murine RPS23RG1 can interact with adenylate cyclase 8 to activate the cAMP/PKA pathway. These results are consistent with previous reports showing that adenylate cyclases are responsible for the synthesis of cAMP^25^, which in turn activates PKA^26^.

PKA mediates phosphorylation of GSK-3α at serine 21 and GSK-3β at serine 9^21^. Consistent with this finding, overexpression of both human *RPS23RG1* and mouse *Rps23rg1* increased levels of inactive phosphorylated GSK-3 in mouse neuroblastoma N2a cells stably expressing the human APP Swedish mutation (N2aSwe), while total GSK-3 levels were unchanged (Fig. 3D). This effect is dependent on PKA activity as the PKA inhibitor H89 abolished p-GSK-3 upregulation induced by *RPS23RG1* overexpression (Fig. 3E). PKA can also phosphorylate CREB at Serine 133, which plays an important role in synaptic and cognitive function^27^,^28^,^29^. As expected, we found that phospho-CREB levels (p-CREB) were also increased upon *RPS23RG1* overexpression (Fig. 3D). In addition to enhancing PKA-mediated phosphorylation, *RPS23RG1* overexpression induced a marked increase in APP βCTF and sAPPα levels (Fig. 3D), implicating a role for *RPS23RG1* in affecting APP cleavage, similar to mouse *Rps23rg1*^22^.

Increased GSK-3 activity contributes greatly to pathophysiological tau hyperphosphorylation in AD^30^. Therefore, it is likely that *RPS23RG1* overexpression can suppress tau phosphorylation by enhancing PKA-mediated GSK-3 phosphorylation/inactivation. To test this, we co-transfected N2aSwe cells with the human tau splice variant T40 together with either human RPS23RG1, mouse Rps23rg1, or control vectors. We then measured tau phosphorylation at serine 396 and serine 404 PHF-1 epitopes representing the major GSK-3 phosphorylation sites found in the paired helical filament component of pathological tangles. While total tau levels were unchanged, tau phosphorylation was significantly reduced in cells transfected with human or mouse Rps23rg1 (Fig. 3F).

Furthermore, we determined whether RPS23RG1 can reduce Aβ production in human HeLa cells expressing the human APP Swedish variant (HeLaSwe) by ELISA. Aβ levels in conditioned media were significantly reduced in HeLaSwe cells transfected with human or mouse RPS23RG1 compared to vector-transfected cells (Fig. 3G). Taken together, our results suggest that human RPS23RG1 activates the AC/cAMP/PKA pathway to enhance CREB activity and inhibit GSK-3 activity, leading to a reduction in tau hyperphosphorylation and Aβ production.

### RPS23RG1 enhances synaptic plasticity and mitigates oAβ-induced synaptic loss

**Figure 4:**
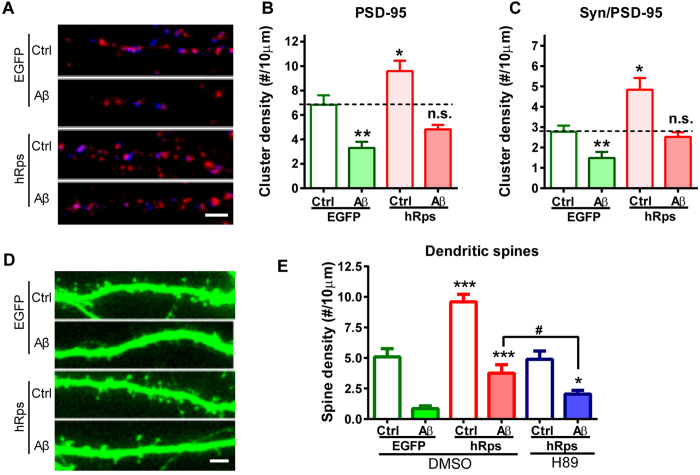
*RPS23RG1*overexpression mitigates oAβ-induced synaptic loss. Cultured neurons were transfected with plasmids encoding human RPS23RG1 (hRps) and EGFP or EGFP alone, and then exposed to oAβs or control Aβ_42-1_. In some experiments, neurons were treated with additional DMSO or the PKA inhibitor H89. (**A**) Immunofluorescence images reveal localization of PSD-95 (red) and synapsin I (Syn; blue) clusters in neuronal dendrites. (**B,C**) Quantification of PSD-95 cluster (**B**) or PSD-95/Syn co-cluster (**C**) densities in neuronal dendrites (n_EGFP/Ctrl_ = 12, n_EGFP/Aβ_ = 10, n_hRps/Ctrl_ = 11, n_hRps/Aβ_ = 13). (D) Dendritic spines were visualized by transfected EGFP. (**E**) Quantification of spine density (n_EGFP/Ctrl/DMSO_ = 11, n_EGFP/Aβ/DMSO_ = 10, n_hRps/Ctrl/DMSO_ = 11, n_hRps/Aβ/DMSO_ = 13, n_hRps/Ctrl/h89_ = 10, n_hRps/Aβ/H89_ = 10). Scale bar, 5 μm. Values were mean ± SEM (n.s., not significant. *^,#^*p* < 0.05, **p < 0.01 one-way ANOVA with Dunnett’s multiple comparisons).

To further characterize a potential neuroprotective function for RPS23RG1, we investigated whether *RPS23RG1* overexpression can prevent oAβ-induced synaptic loss by measuring the density of dendritic spines and synaptic contacts decorated by juxtaposed presynaptic synapsin I and postsynaptic PSD-95 immunoreactive clusters (Syn/PSD-95 co-clusters). To this end, we mimicked pathological stress *in vitro* where cultured murine primary neurons were challenged with soluble oAβs^31^. Soluble oAβs were prepared as described previously^32^, and were a mixture containing about 48% monomer and 52% trimer with a minor fraction of dimer (Supplementary Fig. S2). After overnight transfection with plasmids encoding RPS23RG1-IRES-EGFP or EGFP control, cultured cortical neurons at 21 days *in vitro* (DIV) were incubated with Aβ_1-42_ oligomers (250 nM) or a non-amyloidogenic Aβ_42-1_ control for an additional 24 h. These cells were then processed for immunocytochemistry to evaluate their synaptic integrity. Interestingly, we found that *RPS23RG1* overexpression significantly (*p* < 0.05) increased the density of both PSD-95 clusters and Syn/PSD-95 co-clusters in the absence of oAβ, suggesting that RPS23RG1 can enhance synapses (Fig. 4A–C). On the other hand, oAβ significantly (*p* < 0.01) decreased the densities of both PSD-95 clusters and Syn/PSD-95 co-clusters in EGFP-expressing cells, as well as in *RPS23RG1*-expressing cells (Fig. 4A–C). However, with oAβ treatment and *RPS23RG1* overexpression in combination, we found that both cluster densities in treated neurons were nearly the same as in controls, indicating that *RPS23RG1* overexpression can offset oAβ-induced synaptic loss.

We next determined whether *RPS23RG1* overexpression rescues oAβ-induced dendritic spine loss. We transfected cultured cortical neurons with plasmid vectors encoding EGFP or RPS23RG1-IRES-EGFP. Following overnight transfection, we exposed these cells to 250 nM oAβs or Aβ_42-1_ as a control (Ctrl) for an additional 24 h. Consistent with its role in synaptic enhancement, *RPS23RG1* overexpression caused a significant (*p* < 0.01) increase in dendritic spine density (EGFP/Ctrl, Fig. 4D,E). In contrast, oAβ exposure caused a significant (*p* < 0.01) reduction in dendritic spine density in both EGFP-expressing (EGFP/Aβ vs. EGFP/Ctrl) and hRps-expressing (hRps/Aβ vs. hRps/Ctrl) cells (Fig. 4D,E). However, the density of dendritic spines in hRps/Aβ cells was comparable to that in EGFP/Ctrl cells, and significantly higher than that in EGFP/Aβ cells (Fig. 4D,E), indicating that *RPS23RG1* overexpression mitigated oAβ-induced spine loss. To determine whether RPS23RG1-mediated synaptic protection is PKA-dependent, we treated neuronal cultures with the PKA inhibitor H89 in combination with RPS23RG1 transfection and oAβ application. Upon H89 treatment, dendritic spine density in H89/hRps/Aβ cells was significantly lower than in hRps/Aβ cells, but was not statistically different when compared to EGFP/Aβ cells (Fig. 4E). This demonstrated that H89 abolished the restorative effect of RPS23RG1 on oAβ-induced spine loss. Taken together, these results demonstrate that oAβ-induced synaptic loss can be offset by *RPS23RG1* overexpression in a PKA-dependent manner.

### *Rps23rg1* knockdown aggravates oAβ-induced synaptic loss in cultured mouse neurons

**Figure 5:**
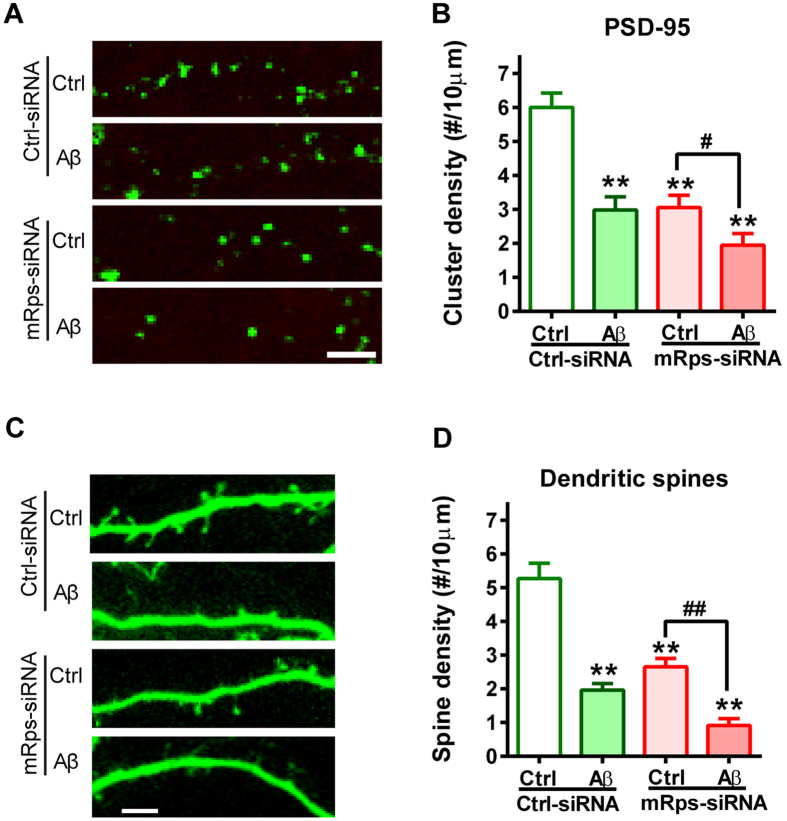
*Rps23rg1*knockdown aggravates oAβ-induced synaptic loss. (**A**) Representative images showing expression of PSD-95 (green) in cultured neurons transfected with *Rps23rg1* or control siRNA and exposed to oAβs or control Aβ_42-1_. Only neuronal dendrites transfected with Cy3-tagged siRNAs (not shown) were selected for analysis. (**B**) Quantification of PSD-95 cluster densities showed that *Rps23rg1* knockdown aggravated oAβ-induced synaptic loss. (**C**) Dendritic spines were visualized by transfected EGFP. (**D**) Graph summary showing that oAβ-induced spine loss was further enhanced by *Rps23rg1* siRNA. Scale bar, 5 μm. Values were mean ± SEM (n = 6 cells per group from 3 cultures). ^#^*p* < 0.05, **^,##^*p* < 0.01 one-way ANOVA with Dunnett’s multiple comparisons.

Next, we used siRNA oligos to downregulate the expression of mouse Rps23rg1 in cultured mouse cortical neurons^22^. Neurons were transfected overnight with either *Rps23rg1* or control siRNAs and exposed to oAβs or control Aβ_42-1_ for an additional 24 h. Cy3-tagged siRNAs were used to visualize siRNA-transfection. In contrast to effects observed with RPS23RG1 overexpression, *Rps23rg1* siRNA application reduced PSD-95 cluster density in the absence of oAβs (Ctrl/Ctrl-siRNA vs. Ctrl/mRps-siRNA) and aggravated oAβ-induced synaptic loss (Ctrl/mRps-siRNA vs. Aβ/mRps-siRNA) (Fig. 5A,B). Meanwhile, *Rps23rg1* siRNA transfection decreased spine density in the absence of oAβs (Ctrl/Ctrl-siRNA vs. Ctrl/mRps-siRNA) and enhanced oAβ-induced spine loss (Ctrl/mRps-siRNA vs. Aβ/mRps-siRNA) (Fig. 5C,D). These results indicate that endogenous RPS23RG1 plays an important role in synaptic maintenance.

### *Rps23rg1* overexpression mitigates oAβ-induced cognitive impairment and synaptic deficits in mice

**Figure 6:**
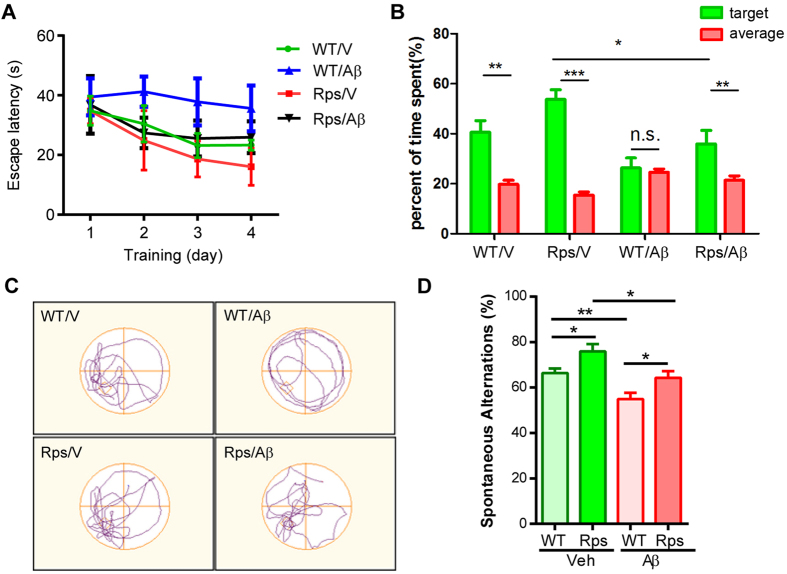
*Rps23rg1* verexpression alleviates oAβ-induced cognitive impairment in mice. (**A**) Summary graph showing latency in finding hidden platforms during training sessions in Morris water maze tests. (**B**) Summary graph showing the percent of time spent in the target and the averaged percent of time spent in the other three quadrants during probe test. (**C**) Representative swimming patterns during probe test were shown. (**D**) Spontaneous alternations in Y-maze test. *Rps23rg1* transgenic (Tg) mice showed improved performance compared to wild type (WT) mice in the presence and absence of oAβs. For both behavior tests, 11 WT/V, 10 WT/Aβ, 13 Tg/V, and 12 Tg/Aβ male mice at 2~3 months old were used, respectively. Values were mean ± SEM (n.s., not significant, *p < 0.05, **p < 0.01, ***p < 0.001 by two-way ANOVA with Dunnett’s multiple comparisons).

To recapitulate results obtained from cultured neurons *in vivo*, we used *Rps23rg1* transgenic (Rps Tg) mice overexpressing mouse *Rps23rg1* with an N-terminal Myc tag under a neuron-specific hThy1 promotor in the brain^22^ to evaluate the effect of *Rps23rg1* overexpression on mitigating oAβ-induced impairment in learning and memory. Rps Tg mice and wild type littermates were bilaterally injected in the hippocampus with oAβs or vehicle control. One week following injection, we evaluated spatial memory performance by Morris water maze tests. During hidden platform training sessions, wild type mice injected with oAβs (WT/Aβ) demonstrated impaired spatial learning as indicated by longer intervals required (*p* < 0.05, two-way ANOVA) to find the hidden platform compared to wild type mice injected with vehicle (WT/V) (Fig. 6A). However, *Rps23rg1* transgenic mice injected with oAβs (Rps/Aβ) showed no difference in platform identification when compared to WT/V or Rps/V mice (Fig. 6A). To determine memory retention, each mouse received one probe test at 24 h after the last training session. By comparing the percent time spent in the target quadrant to the average percent time spent in the other three quadrants, we found that there was memory retention in WT/V (*p* < 0.01), Rps/V (*p* < 0.001), and Rps/Aβ (*p* < 0.01), but not in WT/Aβ mice (Fig. 6B,C). On the other hand, the percent time spent in the target quadrant in Rps/Aβ was lower than that in Rps/V (*p* < 0.05) (Fig. 6B). There were no significant differences in swimming speed and thigmotaxis among all 4 groups of mice (Supplementary Fig. S3 and S4). Following the Morris water maze test, we performed Y-maze test to further evaluate the effect of *Rps23rg1* overexpression on oAβ-induced cognitive impairment. Injection of oAβs caused a significant decrease in spontaneous alternation in both WT (*p* < 0.01) and Rps Tg (*p* < 0.05) mice (Fig. 6D). However, Rps Tg mice always showed significantly more spontaneous alternation than WT mice when treated either with vehicle (*p* < 0.05) or with oAβ (*p* < 0.05) (Fig. 6D). Together, these results demonstrated that although oAβ impairs memory in both WT and Rps Tg mice, *Rps23rg1* overexpression can mitigate oAβ-induced behavioral deficits in learning and memory.

**Figure 7:**
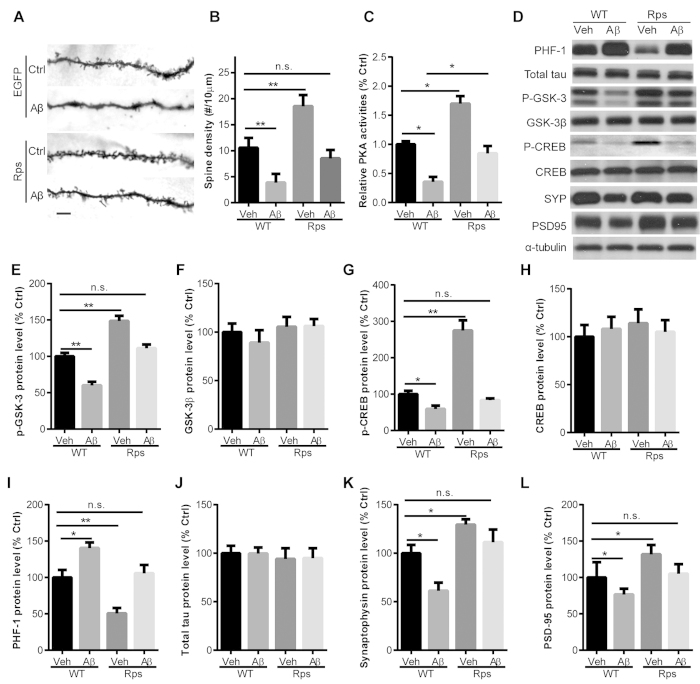
*Rps23rg1* overexpression reduces oAβ-induced synaptic toxicity in mice. (**A**) Golgi staining showing dendritic spines in the hippocampus of wild type (WT) or *Rps23rg1* transgenic (Tg) mice injected with oAβs or vehicle. Scale bar, 5 μm. (**B**) Quantification of spine densities in (**A**). (**C**) *Rps23rg1* overexpression prevented oAβ-induced PKA inactivation. PKA activity was determined in *Rps23rg1* Tg and WT mice injected with oAβs or vehicle in the hippocampus. (**D–L**) Representative gel images (**D**) and summary bar graphs showing expression of p-GSK-3 (**E**), total GSK-3 (**F**), p-CREB (**G**), total CREB (**H**), PHF-1 p-tau (**I**), total tau (**J**), synaptophysin (SYP, K), and PSD-95 (**L**). Values were mean ± SEM (n = 4, n.s., not significant, *p < 0.05, **p < 0.01 one-way ANOVA with Dunnett’s multiple comparisons).

Following behavioral analysis, we performed Golgi staining and western blot analyses in brain tissue derived from the mice described above to determine whether *Rps23rg1* overexpression conferred neuroprotective effects to offset oAβ-induced synaptic loss. By Golgi staining (Fig. 7A), spine density was found to be significantly reduced in WT/Aβ mice (*p* < 0.01) but not in Rps/Aβ mice, when compared to WT/V mice (Fig. 7B). We also found that spine density in *Rps23rg1* Tg mice was significantly higher (*p* < 0.01) than in WT mice (Fig. 7B). These results suggest that *Rps23rg1* overexpression increased spine density under normal conditions and mitigated oAβ-induced spine loss under pathological conditions. Moreover, we found that PKA activity was significantly enhanced in brain tissue lysates from Rps/V mice compared to WT/V mice, and oAβ-induced PKA inactivation was significantly reduced in Rps/Aβ mice compared to WT/Aβ mice (Fig. 7C). These results are consistent with our *in vitro* findings that RPS23RG1-mediated synaptic protection is PKA-dependent (Fig. 4).

By immunoblotting hippocampal lysates, we determined that levels of PKA-mediated phosphorylation on substrates such as p-GSK-3α/β (inactive, Fig. 7D,E) and p-CREB (active, Fig. 7D,G) were significantly higher in *Rps23rg1* Tg mice than in WT/V mice, while total GSK-3β (Fig. 7D,F) and CREB (Fig. 7D,H) levels were unchanged. Under pathological conditions, oAβs induced a significant decrease in p-GSK-3 (Fig. 7E) and p-CREB (Fig. 7G) levels in both WT and *Rps23rg1* Tg mice. However levels of p-GSK-3 and p-CREB were comparable in *Rps23rg1* Tg mice treated with oAβs and WT mice treated with vehicle controls, suggesting that *Rps23rg1* overexpression mitigated oAβ-induced GSK-3 activation or CREB inactivity in mice. Consistent with the notion that aberrant GSK-3 activation leads to tau hyperphosphorylation in AD^30^, phosphorylated tau levels as determined through PHF-1 immunoblotting were significantly increased by oAβ treatment in both WT and *Rps23rg1* Tg mice (Fig. 7D,I). Moreover, oAβ-induced tau-hyperphosphorylation in *Rps23rg1* Tg mice was comparable to that in WT mice treated with vehicles (Fig. 7D,I), whereas no change in total tau levels was observed with oAβ application or *Rps23rg1* overexpression (Fig. 7D,J). Furthermore, expression of synaptic proteins such as synaptophysin and PSD-95, in *Rps23rg1* Tg mice treated with oAβs, was comparable to that in WT mice treated with vehicles (Fig. 7D,K,L). Taken together, these results strongly indicate that oAβ-induced synaptic loss and abnormal protein kinase activity can be ameliorated by *Rps23rg1* overexpression in mice.

### *Rps23rg1* overexpression alleviates oAβ-induced LTP impairment

**Figure 8:**
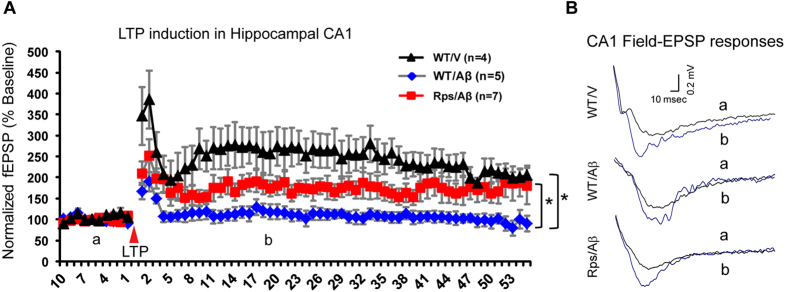
*Rps23rg1*overexpression alleviates oAβ-induced LTP suppression in the hippocampal CA1 region. (**A,B**) Induction of LTP in CA1 was significantly attenuated in wild type (WT) mouse CA1, but not in *Rps23rg1* transgenic (Tg) mouse CA1, following oAβ injection, when compared to WT mice injected with vehicle. In panel (**B**), horizontal line = 10 msec and vertical line = 0.2 mV. LTP was recorded on 4, 5, 7 slices from 3 WT/V, 4 WT/Aβ, and 6 Tg/Aβ mice, respectively. Values were mean ± SEM (*p < 0.05 by two-way ANOVA with Dunnett’s multiple comparisons).

We further examined LTP response to evaluate the effect of *Rps23rg1* overexpression on oAβ-induced synaptic dysfunction. To this end, *Rps23rg1* transgenic or wild type mice were injected with oAβs or vehicle in the hippocampus, where a week later the Schaffer collateral input of the CA1 dendritic region (stratum radiatum) was stimulated at increasing intensities (from minimum to maximum) and field excitatory postsynaptic potential was recorded from the stratum radiatum region. To determine whether *Rps23rg1* overexpression in *Rps23rg1* Tg mice can reduce oAβ-induced LTP suppression in the CA1 region, we applied 2 high frequency train stimuli (100 Hz at 20 sec interval) at half-maximum intensity to Schaffer collateral-CA1 pathways in oAβ- or vehicle-injected mice (Fig. 8C,D). Compared to vehicle-injected wild type controls, oAβ application caused significant attenuation in LTP induction in wild type, but not in *Rps23rg1* transgenic mice (two-way ANOVA; *p* < 0.05, Fig. 8C,D), suggesting that *Rps23rg1* overexpression can attenuate oAβ-induced LTP impairment. This result is consistent with the notion that *Rps23rg1* overexpression can restore biochemical synaptic function and cognitive behavior in the presence of Aβ.

## Discussion

We have identified and characterized the human homolog of mouse *Rps23rg1*, which was previously identified with the ability to reduce Aβ production and tau phosphorylation. We found that human RPS23RG1 functions similarly to its mouse homolog in interacting with adenylate cyclase 8 to activate PKA/CREB and inhibit GSK-3. Remarkably, we also found that overexpression of human *RPS23RG1* mitigated oAβ-induced synaptic loss in a PKA-dependent manner in cultured neurons. Moreover, transgenic mice overexpressing mouse *Rps23rg1* in the brain showed better synaptic function and learning/memory than wild type mice, consistent with a concomitant increase in brain PKA/CREB activity and decreased GSK-3 activity. Although hippocampal injection of oAβs induced synaptic and cognitive dysfunction in both wild type and *Rps23rg1* transgenic mice, *Rps23rg1* transgenic mice injected with oAβs had overall comparable phenotypes to wild type mice injected with vehicle. These results suggest that overexpression of Rps23rg1 can alleviate oAβ-induced neurotoxicity, even though such an effect may not be specific to oAβ but rather through promoting synapse numbers.

PKA plays a pivotal role in spine formation^33^,^34^,^35^ and may mediate synaptic function through the downstream phosphorylation and activation of CREB^27^,^28^,^29^. However, Aβ application has been observed to inhibit PKA activity and PKA-mediated CREB phosphorylation, resulting in decreased CREB activity and LTP inhibition^11^. Additional studies showed that CREB activation rescued spine loss and memory deficits in AD mice^36^ or estradiol-induced spine loss^28^. Interestingly, oAβ-induced LTP suppression and CREB inactivation were reversed by drugs such as rolipram and forskolin which enhance cAMP/PKA-signaling^11^. In this study, we have identified RPS23RG1 as a new PKA activator. Similar to these PKA-activating drugs, *RPS23RG1/Rps23rg1* overexpression can also enhance PKA and downstream CREB activities under normal physiological conditions and restore PKA/CREB activities under pathological conditions. Moreover, through activating PKA signaling pathways, *RPS23RG1/Rps23rg1* overexpression alleviated oAβ-induced synaptic loss, and mitigated oAβ-induced LTP suppression and behavioral deficits in mouse learning and memory. Together, these studies conducted by us and others have suggested that the PKA/CREB signaling pathway plays a pivotal role in oAβ-induced synaptic pathogenesis.

In addition to CREB, GSK-3α/β can also be phosphorylated by PKA^21^. It has been well documented that GSK-3 activity is aberrantly upregulated in AD patients and in animal models, and its upregulation plays a critical role in the pathogenesis of AD^19^,^20^. Moreover, activation of GSK-3β in the absence of Aβ is sufficient to induce dendritic spine loss, which can be prevented by pharmacological GSK-3β inhibition in cultured neurons^37^. In this study, we showed that *Rps23rg1* overexpression enhanced GSK-3β phosphorylation to suppress its activity in cultured N2aSwe cells and in mice injected with oAβs. This effect is likely dependent on PKA activity as the PKA inhibitor H89 abolished *RPS23RG1*-mediated GSK-3β phosphorylation in cultured N2aSwe cells. Aberrantly enhanced GSK-3 activity has been associated with tau hyperphosphorylation in AD^30^. Consistent with its ability to inhibit GSK-3β activity, RPS23RG1 overexpression reduced tau phosphorylation in cultured N2aSwe cells and mitigated oAβ-stimulated tau phosphorylation in *Rps23rg1* Tg mice. Since tau phosphorylation plays an important role in oAβ-induced synaptic loss^14^,^15^, it is possible that *Rps23rg1*-mediated reduction in pathogenic GSK-3 activation and subsequent tau phosphorylation plays a pivotal role in its synaptic protection against oAβs.

In contrast to *Rps23rg1* overexpression, knocking down endogenous *Rps23rg1* enhanced oAβ-dependent synaptic loss as demonstrated by reduced spine and PSD-95 cluster density. Remarkably, *Rps23rg1* knockdown alone may mimic oAβ to induce synaptic loss. Thus, it is likely that endogenous *Rps23rg1* is required in maintaining normal synaptic plasticity. It is also possible that *RPS23RG1* downregulation is involved in synaptic pathogenesis in AD. Consistent with this hypothesis, it was found that *RPS23RG1* mRNA levels were decreased in postmortem brains of AD patients as well as in aged Tg2576 mouse brains. Therefore, it is disease-relevant to counteract oAβ-mediated pathogenesis by overexpressing *RPS23RG1*.

RPS23RG1-mediated synaptic protection against AD was further validated by behavioral and electrophysiological experiments in oAβ-injected *Rps23rg1* Tg or control mice. In both Morris water maze and Y-maze behavioral tests, poor performance induced by hippocampal oAβ-injection was only observed in wild type mice, but not in *Rps23rg1* transgenic mice. These results suggest that *Rps23rg1* overexpression confers resistance to oAβ-induced cognitive impairment in mice. As an electrophysiological paradigm of synaptic plasticity and cognitive function, LTP is impaired in mice expressing elevated Aβ at pathological levels^38^,^39^. Consistent with our behavioral results, *Rps23rg1* overexpression alleviated oAβ-induced LTP impairment in the hippocampal CA1 region. Moreover, *Rps23rg1* overexpression alleviated oAβ-induced spine loss and reductions in synaptophysin and PSD-95.

In summary, we have identified and characterized a human homolog of the mouse *Rps23rg1* gene. In addition to its ability to reduce Aβ production and tau phosphorylation, *Rps23rg1/RPS23RG1* overexpression attenuates oAβ-induced synaptic and cognitive impairments by restoring PKA activity, which subsequently leads to enhanced CREB activity and decreased GSK-3β activity and tau phosphorylation. Since RPS23RG1 levels are found to be decreased in AD, our findings indicate that RPS23RG1 and its downstream pathways may be developed further as potential therapeutic targets in AD, thus providing a new avenue for AD research intended to identify effective cures.

## Methods

### Identification of the human Rps23rg1 homolog

To identify candidate human *Rps23rg1* homologs, we searched the human genome for loci with relatively high homology to the mouse *Rps23rg1* coding sequence. An mRNA sequence corresponding to the identified genomic locus was isolated by nested PCR from a human fetal brain cDNA phagemid library using human chromosome-specific primers and primers designed from phagemid vector. The full-length cDNA sequence was then reconstituted based on the sequence of the resulting PCR product. The cDNA sequence was then confirmed by RT-PCR from human fetal brain total RNA.

### Cell culture

Mouse neuroblastoma N2a cells or human HeLa cells expressing the human APP Swedish mutation (N2aSwe or HeLaSwe) were maintained as described previously^22^. Low- or high-density (5 × 10^3^ or 4 × 10^5 ^cells per 35 mm dish, respectively) primary mouse hippocampal or rat cerebrocortical cultures from E17 embryos of either sex were prepared as previously described^31^,^40^. In short, cerebral cortex or hippocampus was enzymatically dissociated with papain (Collaborative Research) and mechanically dispersed into a single-cell suspension. The dissociated cells were plated onto glass coverslips coated with 0.1 mg/ml poly-L-lysine (Sigma). Neurons were maintained at 37 °C in neural basal medium supplied with B27, 0.5 mM glutamine, and 1X Pen/Strep (Invitrogen).

### Preparation of synthetic Aβ oligomers

Aβ oligomers were prepared using a protocol published previously^32^. In brief, human synthetic Aβ1–42 (Anaspec) was suspended in hexafluoroisopropanol at a concentration of 1 mM and incubated at room temperature for 2 h. The solvent was evaporated with a SpeedVac and resuspended in dry DMSO to a stock concentration of 5 mM, which was kept frozen at −80 °C until use. Aβ monomers were prepared by diluting the stock solution 10-fold in MEM (GIBCO). To oligomerize the Aβ peptide, the stock solution was diluted 10-fold in MEM (GIBCO) and incubated at 4 °C for ≥ 24 h. After brief vortex, the solution was sonicated at 4 °C for 10 min. Both monomeric and oligomeric Aβ peptide solutions were centrifuged on a benchtop centrifuge at 10000 rpm for 2 min. The supernatant was then transferred to a clean tube. The existence of oligomeric Aβ1–42 peptides was confirmed by western blot using 10–20% Novex Tricine gels (Invitrogen), where consequent blots were probed with anti–Aβ antibodies (clone 6E10, Covance).

### Immunocytochemistry

Immunocytochemistry was performed on cultured cells previously described in detail^31^. In brief, cells were rinsed with PBS, fixed with 4% paraformaldehyde (PFA) and permeabilized with 0.1% Triton X-100 in PBS. After blocking with 10% goat serum in PBS, primary antibodies, including anti-PSD-95 mouse monoclonal (NeuroMap) and anti-synapsin I rabbit polyclonal antibodies (Millipore), were applied overnight at 4 °C. After incubation with Alexa Fluor-conjugated secondary antibodies (Invitrogen), cells were fixed and mounted with Fluoromount G (Southern Biotechnology Associates, Inc.). Images were captured by deconvolution microscopy and analyzed with SlideBook 5.5 software (Intelligent Imaging Innovations) or NIH ImageJ 1.45s. Clusters were scored for PSD-95 along primary and secondary dendrites over a dendritic length of at least 30 μm. The density of these clusters was determined as the number of clusters per 10 μm for each neuron. Similarly, the number of PSD-95 clusters juxtaposed to synapsin I were also determined. Statistical significance was determined by one-way ANOVA with post-hoc tests (for multiple comparisons) or Student’s *t*-test (for two-way comparisons).

### Quantification of dendritic spines

To visualize dendritic spines, cells were transduced with plasmid or lentiviral vectors carrying EGFP^40^,^41^. After EGFP expression was confirmed, cells were fixed with 4% PFA and mounted with Flouromount G. Images were captured by deconvolution microscopy and analyzed for dendritic spine density. We counted the number of dendritic spines along primary and secondary dendrites on projected deconvolved images, typically along a 30 μm or longer distance. Densities of dendritic spines are scored as the number of spines per 10 μm. Statistical significance was determined by one-way ANOVA with post-hoc tests (for multiple comparisons) or Student’s *t*-test (for two-way comparisons).

### Co-immunoprecipitation and immunoblotting

Co-immunoprecipitation experiments were performed as described previously^22^. In brief, N2aSwe cells transfected with mouse Rps23rg1, human RPS23RG1, or control vectors were lysed in either CHAPSO buffer (1% CHAPSO, 25 mM HEPES [pH 7.4], 150 mM NaCl, and 2 mM EDTA supplemented with protease inhibitors) or in NP40 buffer (1% Non-Idet P40 in phosphate buffered saline, supplemented with protease inhibitors). Lysates were immunoprecipitated using mouse IgG, rabbit IgG, and antibodies against adenylate cyclase 8 (AC8) and Trueblot IP beads (eBioscience), followed by western blot with antibodies against Myc. Co-IP inputs were also subjected to western blot with antibodies against AC8 or Myc.

### Aβ ELISA assay

HeLaSwe cells were transfected with mouse Rps23rg1 human RPS23RG1, or control vectors. Conditioned media and lysates from these cells were collected. Aβ1-42 levels were quantified using ELISA kits (Invitrogen), following the manufacturer’s protocols.

### *In vitro* PKA activity

PKA activity was assayed using a commercial kit (Upstate), following the manufacturer’s protocol.

### Pharmacological treatments with the PKA inhibitor H89

As described previously^22^, N2aSwe cells were transfected with mouse Rps23rg1, human RPS23RG1, or control vectors, and then split equally. Four hours prior to collection, cells were treated with the PKA inhibitor H89 (10 mM) or with DMSO. Cells were then collected for western blot analysis. For primary cultures, cultured neurons were transfected with human RPS23RG1 or control vectors. After overnight transfection, cells were treated with H89 (10 mM) or DMSO and exposed to Aβ oligomers (250 nM) or vehicle simultaneously for an additional 24 h. The cells were then fixed and processed for dendritic spine density analysis.

### *Rps23rg1* RNA interference

*Rps23rg1* knockdown experiments using *Rps23rg1* siRNA sequences were previously described in detail^22^. Cultured neurons were transfected with *Rps23rg1* or control siRNA using Lipofectamine RNAiMAX reagent (Invitrogen), following the manufacturer’s protocol. After *Rps23rg1* RNA interference, the density of PSD-95 clusters and Syn/PSD-95 co-clusters were determined as described above.

### Mice and *in vivo* analysis

Tg2576 mice (in B6:SJL background) expressing human APP695 “Swedish” allele (KM670/671NL)^24^ were originally purchased from Taconic and bread in house. Tg2576 mice, wild type B6 mice and the transgenic mice (congenic in B6 background) expressing mouse *Rps23rg1* with a neuron-specific human Thy1 promotor^22^ were maintained at the Animal Facility of Sanford-Burnham-Prebys Medical Discovery Institute. The temperature was maintained at 22 ± 2 °C at 65 ± 6% humidity. Animals were kept at 12 hours night and day cycle with free access to water and normal chow diet. All procedures for maintaining and using mice described in this study were in accordance with relevant guidelines of the Animal Welfare Act and the DHHS “Guide for the Care and Use of Laboratory Animals”, and approved by the Institutional Animal Care and Use Committee of Sanford-Burnham-Prebys Medical Discovery Institute. Only male mice at 2–3 months were used in this study. Care was taken to reduce the suffering of the animals during experiments.

### Stereotactic injection of Aβ oligomers

The dosage and treatment time were based on a previous publication^42^. Briefly, Aβ_1-42_ oligomers (1.5 μL, 20 μM) or equivalent vehicle controls were stereotactically injected (at 0.5μL/min) into the hippocampus of the brain-specific *Rps23rg1* transgenic or wild type mice at the following coordinates: anterior posterior, −2.0 mm; medial lateral, ±1.3 mm; dorsal ventral, 2.1 mm. One week after injection, mice were used for behavioral tests, electrophysiological recordings, immunohistochemistry (Golgi staining), or immunoblot analysis as described.

### Morris water maze test

A version of the conventional Morris water maze test (San Diego Instruments) was performed to evaluate spatial reference learning and memory^39^. Mice were trained to escape to an invisible platform submerged 1.5 cm beneath the water surface 14 cm in diameter. If a mouse failed to find the platform within 60 s, it was manually guided to the platform and allowed to remain there for 10 s. The escape latency was scored as 60 s for these mice. The swim speed and the time that an animal spent swimming within 15 cm of the pool wall (thigmotaxis)^43^ were also recorded. Mice were given 4 trials per day and retention of spatial training (probe test) was assessed 24 h following the last training trial. Each mouse received only one probe test that consisted of a 60 s free swim in the pool without the platform. The time spent in each quadrant was recorded. The target quadrant is defined by the location where the hidden platform was previously placed in the hidden training sessions, but removed during the probe test. The ANY-maze video tracking system (Stoelting Co.) was used to record all trials for automated analysis.

### Y maze test

Y-maze test was performed to evaluate working memory. Each mouse was placed in the center of the Y maze. A single 5 min test was performed and recorded by video camera. Arm entries and the order of entries are determined on recorded video. Spontaneous alternations are defined as consecutive triplets of different arm choices.

### Golgi staining

FD Rapid GolgiStain Kit (FD NeuroTechonologies) was used for Golgi staining according to vendor’s protocol to determine dendritic spine density in oAβ- or vehicle-injected *Rps23rg1 *mice and wild type littermate controls. Images were acquired using a Zeiss fluorescence microscope using a 40× objective under differential interference contrast (DIC). Dendritic spine density was measured using NIH ImageJ 1.45s.

### Quantitative real-time PCR

Total RNA was extracted from brain tissues obtained from Tg2576 mice^24^ or control littermates. Following first strand synthesis, mRNA concentrations were determined by real-time PCR using an iCycler iQ with SYBR green supermix (Bio-Rad). The primer pair used for real-time PCR was: Rps23rg1-5′ (5′-TGTTGCATACACATACATGC-3′) and Rps23rg1-3′ (5′-TCATTAAGAACGG GAAGAAG-3′). β-actin primers served as controls^22^.

### Electrophysiological recordings

One week after oAβ injection, male *Rps23rg1* transgenic and wild type littermate mice^22^ at 9 weeks old were anesthetized with isoflurane. The mice were then decapitated and their brains were dissected immediately. Using a slicing vibrotome, 300 μm transverse hippocampal sections was collected in cold (2–4 ^o^C), oxygenated (95% O_2_, 5% CO_2_) cutting artificial cerebrospinal fluids (cutting-ACSF) and the CA3 region of hippocampus was removed. The cutting-ACSF contained (in mM): 246 Sucrose, 1.25 NaH_2_PO_4_, 2 KCl, 26 NaHCO_3_, 10 glucose, 1 Na-L-Ascorbate, 3 MgCl_2_ (pH = 7.4, Osm = 300 mOsmole). The sections were then transferred to oxygenated ACSF and allowed to equilibrate for at least 1 h in a humidified water bath at (28–30 ^o^C). The ACSF contained (in mM): 130 NaCl, 24 NaHCO_3_, 10 glucose, 1.5 MgSO_4_.7H_2_O, 1.25 NaH_2_PO_4_, 3.5 KCl, 2 CaCl_2_ (pH = 7.4, Osm = 315–330 mOsmole). An acute hippocampal slice was then transferred to a recording chamber and secured using a slice-anchoring harp. Schaffer collateral inputs to the CA1 region was stimulated with a bipolar tungsten electrical stimulating electrode at various increasing intensities. Using a low resistance recording electrode (1–3 megaOhm) filled with ACSF, field Excitatory Postsynaptic Potential (f EPSP) responses from the CA1 stratum radiatum region was recorded using a MultiClamp 700B (Axon Instrument). The initial slope of fEPSP response was measured using Clampex software. Synaptic transmission of CA1 neurons was determined as input-ouput curves for fEPSP slope response to Schaffer collateral stimulation.

LTP was induced after establishing stable baseline fEPSP recordings for 10–15 m. LTP induction consisted of two high frequency trains (100 Hz - HFTs) stimuli (at 20 s intervals) at 40–50% of the maximum stimulus intensity. Recording of the CA1 region was resumed immediately after LTP induction for a total duration of 60 minutes. The initial slope of the fEPSP was measured and normalized to baseline recordings. Data analysis was carried out offline using Clampex Software and two-way ANOVA with post hoc tests was used to establish the statistical significance between data sets.

### Statistical analysis

Data were presented as mean ± SEM. All data were analyzed using Prism 6 software (GraphPad Software, Inc.) unless otherwise indicated. For Quantitative real-time PCR data, statistical significance was determined by Student’s *t* test for pairwise comparisons. For western blot, Golgi staining and immunofluorescence data, statistical significance was determined by one-way ANOVA with Dunnett’s multiple comparisons. For behavior test and LTP data, statistical significance was determined by two-way ANOVA with Dunnett’s multiple comparisons. *p* < 0.05 was considered statistically significant.

## Additional Information

**How to cite this article**: Yan, L. *et al.* RPS23RG1 reduces Aβ oligomer-induced synaptic and cognitive deficits. *Sci. Rep.* **6**, 18668; doi: 10.1038/srep18668 (2016).

## Supplementary Material

Supplementary Information

## References

[b1] Li Z. & Sheng M. Caspases in synaptic plasticity. Mol Brain 5, 15 (2012). 22583788 10.1186/1756-6606-5-15 PMC3366905

[b2] Tu S., Okamoto S. I., Lipton S. A. & Xu H. Oligomeric Aβ-induced synaptic dysfunction in Alzheimer’s disease. Mol Neurodegener 9, 48 (2014). 25394486 10.1186/1750-1326-9-48 PMC4237769

[b3] Terry R. D. *et al.* Physical basis of cognitive alterations in alzheimer’s disease: Synapse loss is the major correlate of cognitive impairment. Ann Neurol 30, 572–580 (1991). 1789684 10.1002/ana.410300410

[b4] Zemlan F. P. *et al.* Quantification of axonal damage in traumatic brain injury: affinity purification and characterization of cerebrospinal fluid tau proteins. J Neurochem 72, 741–50 (1999). 9930748 10.1046/j.1471-4159.1999.0720741.x

[b5] Kouri N. *et al.* Genome-wide association study of corticobasal degeneration identifies risk variants shared with progressive supranuclear palsy. Nat Commun 6, 7247 (2015). 26077951 10.1038/ncomms8247 PMC4469997

[b6] Jiang S. *et al.* Trafficking regulation of proteins in Alzheimer’s disease. Mol Neurodegener 9, 6 (2014). 24410826 10.1186/1750-1326-9-6 PMC3891995

[b7] Zheng H. & Koo E. H. Biology and pathophysiology of the amyloid precursor protein. Mol Neurodegener 6, 27 (2011). 21527012 10.1186/1750-1326-6-27 PMC3098799

[b8] Shankar G. M. *et al.* Amyloid-beta protein dimers isolated directly from Alzheimer’s brains impair synaptic plasticity and memory. Nat Med 14, 837–42 (2008). 18568035 10.1038/nm1782 PMC2772133

[b9] Kandel E. R. The molecular biology of memory: cAMP, PKA, CRE, CREB-1, CREB-2, and CPEB. Mol Brain 5, 14 (2012). 22583753 10.1186/1756-6606-5-14 PMC3514210

[b10] Kim S. H., Nairn A. C., Cairns N. & Lubec G. Decreased levels of ARPP-19 and PKA in brains of Down syndrome and Alzheimer’s disease. J Neural Transm Suppl, 263–72 (2001). 11771749 10.1007/978-3-7091-6262-0_21

[b11] Vitolo O. V. *et al.* Amyloid beta -peptide inhibition of the PKA/CREB pathway and long-term potentiation: reversibility by drugs that enhance cAMP signaling. Proc Natl Acad Sci USA 99, 13217–21 (2002). 12244210 10.1073/pnas.172504199 PMC130613

[b12] Gong B. *et al.* Ubiquitin hydrolase Uch-L1 rescues β-amyloid-induced decreases in synaptic function and contextual memory. Cell 126, 775–788 (2006). 16923396 10.1016/j.cell.2006.06.046

[b13] Zhao X. L. *et al.* Expression of beta-amyloid Induced age-dependent presynaptic and axonal changes in Drosophila. J Neurosci 30, 1512–22 (2010). 20107079 10.1523/JNEUROSCI.3699-09.2010 PMC6633795

[b14] Ballatore C., Lee V. M. & Trojanowski J. Q. Tau-mediated neurodegeneration in Alzheimer’s disease and related disorders. Nat. Rev. Neurosci. 8, 663–72 (2007). 17684513 10.1038/nrn2194

[b15] Naj A. C. *et al.* Common variants at MS4A4/MS4A6E, CD2AP, CD33 and EPHA1 are associated with late-onset Alzheimer’s disease. Nat Genet 43, 436–41 (2011). 21460841 10.1038/ng.801 PMC3090745

[b16] Zempel H., Thies E., Mandelkow E. & Mandelkow E. M. Aβ oligomers cause localized Ca^(2+)^ elevation, missorting of endogenous Tau into dendrites, Tau phosphorylation, and destruction of microtubules and spines. J Neurosci 30, 11938–50 (2010). 20826658 10.1523/JNEUROSCI.2357-10.2010 PMC6633549

[b17] De Felice F. G. *et al.* Alzheimer’s disease-type neuronal tau hyperphosphorylation induced by Aβ oligomers. Neurobiol Aging 29, 1334–47 (2008). 17403556 10.1016/j.neurobiolaging.2007.02.029 PMC3142933

[b18] Jin M. *et al.* Soluble amyloid β-protein dimers isolated from Alzheimer cortex directly induce Tau hyperphosphorylation and neuritic degeneration. Proc Natl Acad Sci USA 108, 5819–5824 (2011). 21421841 10.1073/pnas.1017033108 PMC3078381

[b19] Frame S. & Cohen P. GSK3 takes centre stage more than 20 years after its discovery. Biochem J 359, 1–16 (2001). 11563964 10.1042/0264-6021:3590001 PMC1222116

[b20] Llorens-Martin M., Jurado J., Hernandez F. & Avila J. GSK-3β, a pivotal kinase in Alzheimer disease. Front Mol Neurosci 7, 46 (2014). 24904272 10.3389/fnmol.2014.00046 PMC4033045

[b21] Fang X. *et al.* Phosphorylation and inactivation of glycogen synthase kinase 3 by protein kinase A. Proc Natl Acad Sci USA 97, 11960–5 (2000). 11035810 10.1073/pnas.220413597 PMC17277

[b22] Zhang Y. W. *et al.* A functional mouse retroposed gene Rps23r1 reduces Alzheimer’s β-amyloid levels and tau phosphorylation. Neuron 64, 328–40 (2009). 19914182 10.1016/j.neuron.2009.08.036 PMC3846276

[b23] Huang X. *et al.* The Rps23rg gene family originated through retroposition of the ribosomal protein s23 mRNA and encodes proteins that decrease Alzheimer’s β-amyloid level and tau phosphorylation. Hum Mol Genet 19, 3835–43 (2010). 20650958 10.1093/hmg/ddq302 PMC2935860

[b24] Hsiao K. *et al.* Correlative memory deficits, Abeta elevation, and amyloid plaques in transgenic mice. Science 274, 99–102 (1996). 8810256 10.1126/science.274.5284.99

[b25] Kamenetsky M. *et al.* Molecular details of cAMP generation in mammalian cells: a tale of two systems. J Mol Biol 362, 623–39 (2006). 16934836 10.1016/j.jmb.2006.07.045 PMC3662476

[b26] Taylor S. S. *et al.* Signaling through cAMP and cAMP-dependent protein kinase: diverse strategies for drug design. Biochim Biophys Acta 1784, 16–26 (2008). 17996741 10.1016/j.bbapap.2007.10.002 PMC2561045

[b27] Gonzalez G. A. & Montminy M. R. Cyclic AMP stimulates somatostatin gene transcription by phosphorylation of CREB at serine 133. Cell 59, 675–680 (1989). 2573431 10.1016/0092-8674(89)90013-5

[b28] Segal M. & Murphy D. D. CREB activation mediates plasticity in cultured hippocampal neurons. Neural Plast 6, 1–7 (1998). 9920677 10.1155/NP.1998.1 PMC2565317

[b29] Delghandi M. P., Johannessen M. & Moens U. The cAMP signalling pathway activates CREB through PKA, p38 and MSK1 in NIH 3T3 cells. Cell Signal 17, 1343–1351 (2005). 16125054 10.1016/j.cellsig.2005.02.003

[b30] Flaherty D. B., Soria J. P., Tomasiewicz H. G. & Wood J. G. Phosphorylation of human tau protein by microtubule-associated kinases: GSK3beta and cdk5 are key participants. J Neurosci Res 62, 463–72 (2000). 11054815 10.1002/1097-4547(20001101)62:3<463::AID-JNR16>3.0.CO;2-7

[b31] Nakanishi N. *et al.* Synaptic protein α1-takusan mitigates amyloid-β-induced synaptic loss via interaction with tau and postsynaptic density-95 at postsynaptic sites. J Neurosci 33, 14170–14183 (2013). 23986251 10.1523/JNEUROSCI.4646-10.2013 PMC3756761

[b32] Molokanova E. *et al.* Differential effects of synaptic and extrasynaptic NMDA receptors on Aβ-induced nitric oxide production in cerebrocortical neurons. J Neurosci 34, 5023–5028 (2014). 24695719 10.1523/JNEUROSCI.2907-13.2014 PMC3972726

[b33] Lin Y.-L., Lei Y.-T., Hong C.-J. & Hsueh Y.-P. Syndecan-2 induces filopodia and dendritic spine formation via the neurofibromin–PKA–Ena/VASP pathway. J Cell Biol 177, 829–841 (2007). 17548511 10.1083/jcb.200608121 PMC2064283

[b34] Yang Y., Wang X.-b., Frerking M. & Zhou Q. Spine expansion and stabilization associated with long-term potentiation. J Neurosci 28, 5740–5751 (2008). 18509035 10.1523/JNEUROSCI.3998-07.2008 PMC2561912

[b35] Zhang X.-l. *et al.* Essential role for synaptopodin in dendritic spine plasticity of the developing hippocampus. J Neurosci 33, 12510–12518 (2013). 23884954 10.1523/JNEUROSCI.2983-12.2013 PMC3721850

[b36] Yiu A. P., Rashid A. J. & Josselyn S. A. Increasing CREB function in the CA1 region of dorsal hippocampus rescues the spatial memory deficits in a mouse model of Alzheimer’s disease. Neuropsychopharmacology 36, 2169–86 (2011). 21734652 10.1038/npp.2011.107 PMC3176558

[b37] DaRocha-Souto B. *et al.* Activation of glycogen synthase kinase-3 β mediates β-amyloid induced neuritic damage in Alzheimer’s disease. Neurobiol Dis 45, 425–37 (2012). 21945540 10.1016/j.nbd.2011.09.002 PMC3694284

[b38] Oddo S. *et al.* Triple-transgenic model of Alzheimer’s disease with plaques and tangles: intracellular Abeta and synaptic dysfunction. Neuron 39, 409–21 (2003). 12895417 10.1016/s0896-6273(03)00434-3

[b39] Billings L. M., Oddo S., Green K. N., McGaugh J. L. & LaFerla F. M. Intraneuronal Abeta causes the onset of early Alzheimer’s disease-related cognitive deficits in transgenic mice. Neuron 45, 675–88 (2005). 15748844 10.1016/j.neuron.2005.01.040

[b40] Nakanishi N. *et al.* Neuroprotection by the NR3A Subunit of the NMDA Receptor. J Neurosci 29, 5260–5265 (2009). 19386922 10.1523/JNEUROSCI.1067-09.2009 PMC2703294

[b41] Tu S. *et al.* Takusan: A large gene family that regulates synaptic activity. Neuron 55, 69–85 (2007). 17610818 10.1016/j.neuron.2007.06.021 PMC2902460

[b42] Moon M. *et al.* Ghrelin ameliorates cognitive dysfunction and neurodegeneration in intrahippocampal amyloid-beta1-42 oligomer-injected mice. J Alzheimers Dis 23, 147–59 (2011). 20930280 10.3233/JAD-2010-101263

[b43] Fridgeirsdottir G. A., Hillered L. & Clausen F. Escalated handling of young C57BL/6 mice results in altered Morris water maze performance. Ups J Med Sci 119, 1–9 (2014). 24172203 10.3109/03009734.2013.847511 PMC3916711

