# Supplementary material for: RPS23RG1 reduces Aβ oligomer-induced synaptic and cognitive deficits
Source: Sci Rep. 2016 Jan 6;6:18668. doi: 10.1038/srep18668 (PMC4702092; doi:10.1038/srep18668)
Supplement: Supplementary Information [file srep18668-s1.doc]

**Supplementary Material**

**RPS23RG1 reduces Aβ oligomer-induced synaptic and cognitive deficits**

Li Yan^1,2,*^, Yaomin Chen^2,*^, Wubo Li^3,*^, Xiumei Huang^2^, Hedieh Badie^2^, Fan Jian^2^, Timothy Huang^2^, Yingjun Zhao^2^, Stanley N. Cohen^4^, Limin Li^3^, Yun-wu Zhang^2,5^, Huanmin Luo^1^, Shichun Tu^2^ & Huaxi Xu^2^

^1^Department of Pharmacology, School of Medicine, Jinan University, Guangzhou 510632, China, ^2^Neuroscience and Aging Research Center, Sanford-Burnham-Prebys Medical Discovery Institute, La Jolla, CA 92037, USA, ^3^Functional Genetics, Inc., Gaithersburg, MD, USA, ^4^Department of Genetics, Stanford University School of Medicine, Stanford, CA 94305, USA, ^5^Fujian Provincial Key Laboratory of Neurodegenerative Disease and Aging Research, Institute of Neuroscience, College of Medicine, Xiamen University, Xiamen 361102, China

**Table S1. Information regarding AD patients and controls used in this study**

| Case | Gender | Age | Braak stage |
| --- | --- | --- | --- |
| Controls |  |  |  |
| 97-17^1^ | M | 78 |  |
| 98-32^1^ | M | 83 |  |
| 99-25^1^ | F | 76 |  |
| 00-49^1^ | F | 86 |  |
| 5130^1^ | M | 71 |  |
| 5049 | F | 102 |  |
| 5105^1^ | F | 74 |  |
| 4996^1^ | M | 91 |  |
| AD patients |  |  |  |
| 99-15 | M | 83 | VI |
| 99-39 | M | 72 | V |
| 01-05 | M | 88 | V/VI |
| 01-09 | F | 83 | V |
| 01-17 | F | 78 | V |
| 03-02 | M | 76 | V/VI |
| X5440 | F | 73 | n.a. |
| X5566 | F | 84 | n.a. |
| PD patients |  |  |  |
| 5412 | M | 77 | - |
| 5287 | M | 96 | - |
| 4908 | M | 78 | - |
| 5003 | F | 81 | - |
| 5244 | F | 82 | - |
| 5532 | M | 75 | - |
| 5572 | M | 93 | - |

^1^Used as controls for comparison with PD patients.

n.a.: not available.


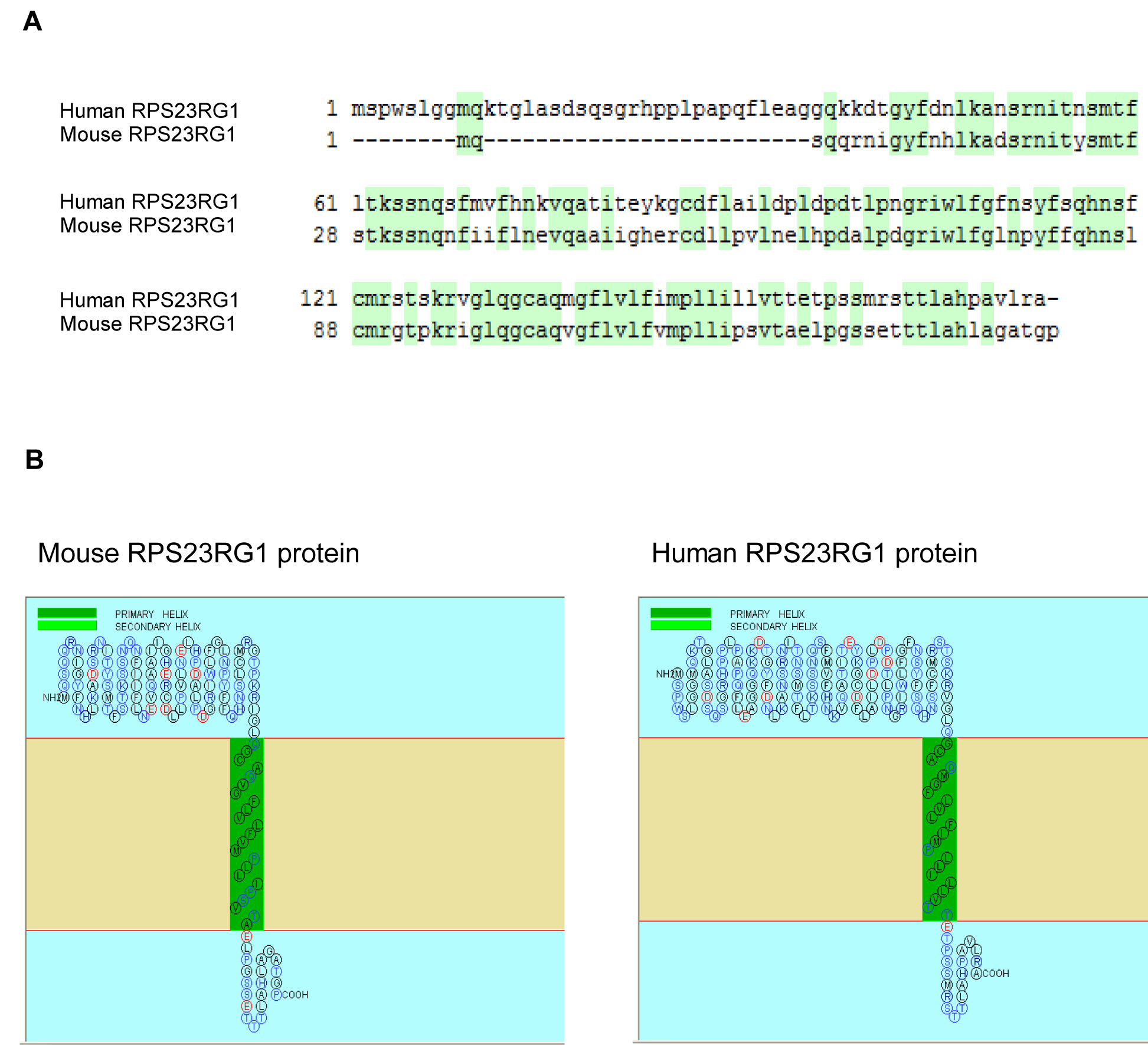


**Supplementary Figure S1. Comparison of human and mouse RPS23RG1 proteins.** (A) Sequence alignment of human and mouse PRS23RG1 proteins. (B) RPS23RG1 sequences from human and mouse were compared using SOSUI to predict transmembrane regions. Sequences in the middle panel indicate transmembrane domains. Upper residues are extracellular and bottom residues represent intracellular portions.


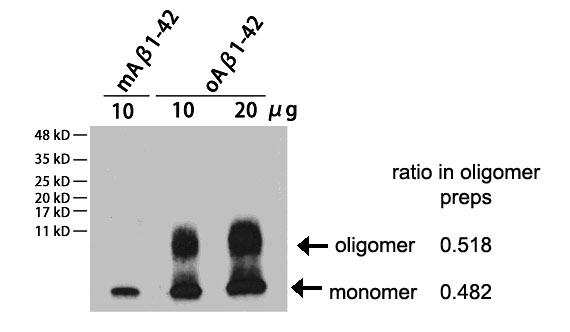


**Supplementary Figure S2. Immunoblot of monomeric and oligomeric synthetic Aβ1–42 peptide preparations under denaturing (SDS) conditions.** Monomeric (Left, 10 µg) or oligomeric (middle 10 µg or right 20 µg) Aβ1–42 peptides were electrophoresed and probed with anti–Aβ antibodies (clone 6E10). In the monomeric Aβ1-42 lane (mAβ1-42), only one predominant immunoreactive band corresponding to the size of Aβ monomer was present. In contrast, the oligomeric Aβ preparation (oAβ1-42) exhibited multiple bands corresponding to the size of Aβ monomers, dimers, and trimers. Molecular weight markers (kDa) are shown at left. The ratio of oligomers to monomers in oligomeric oAβ1-42 was measured to be 0.518 (oligo) to 0.482 (monomer) in oligomeric preparations as determined through quantification of relative band densities.


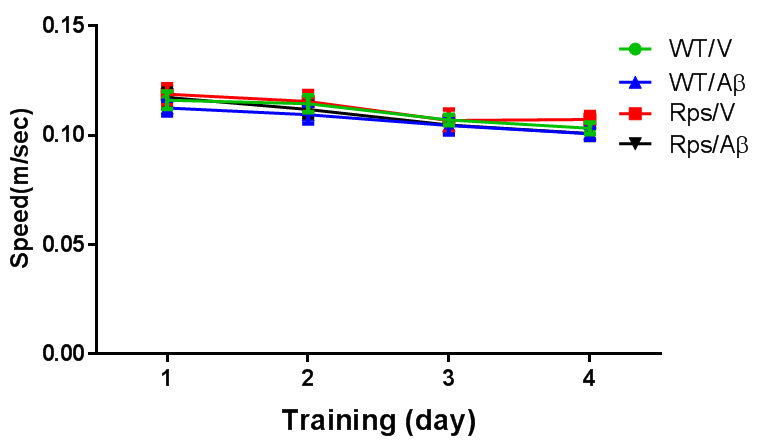


**Supplementary Figure S3. *Rps23rg1* overexpression and oAβ-exposure has no effect on swimming speed in mice in Morris water maze tests.** Summary graph showing swimming speed during training sessions in Morris water maze tests. Values represent mean ± SEM. 11 WT/V, 10 WT/Aβ, 13 Tg/V, and 12 Tg/Aβ male mice at 2~3 months old were used, respectively.


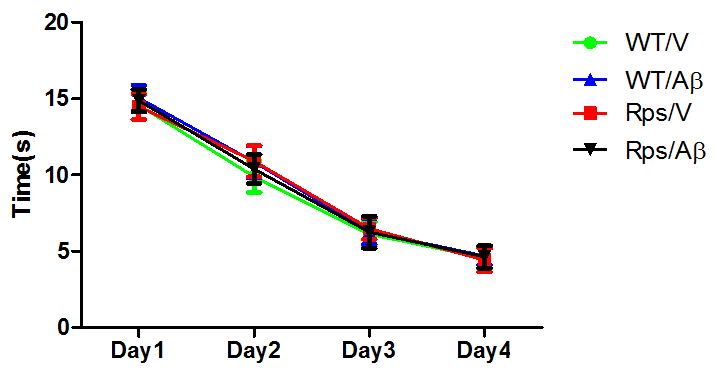


**Supplementary Figure S4. *Rps23rg1* overexpression and oAβ-exposure does not affect mouse thigmotaxis in Morris water maze tests.** Summary graph showing time spent within 15 cm of the pool wall during training sessions in Morris water maze tests. Values represent mean ± SEM. 11 WT/V, 10 WT/Aβ, 13 Tg/V, and 12 Tg/Aβ male mice at 2~3 months old were used, respectively.
